# Delayed onset of changes in soma action potential genesis in nociceptive A-beta DRG neurons in vivo in a rat model of osteoarthritis

**DOI:** 10.1186/1744-8069-5-57

**Published:** 2009-09-28

**Authors:** Qi Wu, James L Henry

**Affiliations:** 1 Michael G DeGroote Institute for Pain Research and Care, McMaster University, 1200 Main Street West, HSC 4N35, Hamilton ON, L8N 3Z5, Canada

## Abstract

**Background:**

Clinical data on osteoarthritis (OA) suggest widespread changes in sensory function that vary during the progression of OA. In previous studies on a surgically-induced animal model of OA we have observed that changes in structure and gene expression follow a variable trajectory over the initial days and weeks. To investigate mechanisms underlying changes in sensory function in this model, the present electrophysiological study compared properties of primary sensory nociceptive neurons at one and two months after model induction with properties in naïve control animals. Pilot data indicated no difference in C- or Aδ-fiber associated neurons and therefore the focus is on Aβ-fiber nociceptive neurons.

**Results:**

At one month after unilateral derangement of the knee by cutting the anterior cruciate ligament and removing the medial meniscus, the only changes observed in Aβ-fiber dorsal root ganglion (DRG) neurons were in nociceptor-like unresponsive neurons bearing a hump on the repolarization phase; these changes consisted of longer half width, reflecting slowed dynamics of AP genesis, a depolarized Vm and an increased AP amplitude. At two months, changes observed were in Aβ-fiber high threshold mechanoreceptors, which exhibited shorter AP duration at base and half width, shorter rise time and fall time, and faster maximum rising rate/maximum falling rate, reflecting accelerated dynamics of AP genesis.

**Conclusion:**

These data indicate that Aβ nociceptive neurons undergo significant changes that vary in time and occur later than changes in structure and in nociceptive scores in this surgically induced OA model. Thus, if changes in Aβ-fiber nociceptive neurons in this model reflect a role in OA pain, they may relate to mechanisms underlying pain associated with advanced OA.

## Background

Osteoarthritis (OA) afflicts an estimated 12-27% of adults over the age of 26 [1] and is characterized by alterations in sensory function, including pain [2,3]. Recently, a multi-center study led by Hawker et al. (2008) revealed two distinct types of OA pain: an early predictable dull, aching, throbbing "background" pain and an unpredictable short episode of intense pain that develops later [4]. During the progression of OA, pain evolves from the "background" pain that is use-related in early OA [5]. Later, this evolves into unpredictable short episodes of intense pain on top of the "background" pain in advanced OA. It is this unpredictable intense pain that has the greatest impact on the quality of life and that results in the avoidance of social and recreational activities [4]. Chronicity of OA [6] suggests that this is a progressive disorder that develops longitudinally in time.

In addition to this clinical evidence, further evidence from animal models of OA support the idea that nociception varies longitudinally and, as a result, different mechanisms may come into play at different times. For example, to address mechanisms underlying these functional changes in OA we have been studying an animal model of OA that exhibits changes in cartilage and bone closely matching the human condition, including cartilage edema and collage turnover [7,8] and that demonstrates significant changes in gene expression of joint tissues [9]. We have found that development of the typical changes that are observed may follow a variable trajectory [10]. Some changes occur early but subside later in model development, including genes in the chemokine, endothelin and epidermal growth factor signaling pathways [7,9]. Further, a recent study done in a surgically-induced OA model in the guinea pig has reported an augmentation in the joint movement-evoked discharge selectively in C-fibers at one week after model induction and in Aδ neurons at one day, one week and three weeks after model induction [11]. Importantly, the change in C-fibers is transient, and reverses by three weeks.

The fact that there is a progression of the pain and of nociceptive signals raises the possibility of a succession of mechanisms involved in changes in sensory function. Among the sites to investigate changes in the neural substrate of nociception are the dorsal root ganglia (DRG), which contain the cell bodies of primary sensory neurons that project from the periphery to the spinal cord. With the idea that a change in sensitivity or function of primary afferent neurons is reflected in the configuration of the action potential (AP) in these neurons, we undertook a study to determine whether changes occur in DRG neurons following induction of OA in our rat model, whether changes observed followed any particular time course of development, and whether changes were associated with a particular functional type of neuron. Several proposals have been made previously to account for the pain of OA, such as activation of sensitized nociceptive neurons in the knee [2,12,13]. Nociceptive primary sensory neurons are those having receptive endings with a high stimulus threshold and that respond preferentially to noxious stimuli [14]. These nociceptive neurons actually conduct in all three velocity ranges of sensory neurons, C-, Aδ- and Aα/β, but in many studies are often considered to conduct only in the C- or Aδ-range of velocities, and Aα/β primary sensory neurons are generally thought to be only non-nociceptive. Our pilot data, however, did not reveal significant changes in AP configuration in C- or Aδ-fiber associated neurons, yet changes were seen in Aβ neurons [15].

A perusal of the literature indicates that there is a considerable number of nociceptive neurons that conduct in the Aβ range: approximately 12% of A-fibers innervating hairy skin in the monkey [16], 20% of Aβ-fibers in cats [17,18] and 30% of Aβ-fibers in rodents [19-22]. High threshold mechanoreceptors are the main type of A-fiber nociceptor, and the other two less common types are mechano-heat nociceptors, and mechano-cold nociceptors [16,23]. Moreover, A-fiber neurons have been suggested to be involved in models of chronic pain [24,25]. Therefore, the present study was done to identify any changes specifically in high threshold Aβ-fiber neurons, and to determine whether there is a progression of change in A-fiber nociceptors through early and later stages of the progression of the model. The present OA model was designed to mimic the most prevalent etiology in human knee OA, which is destabilization of the joint due to an injury [26]. Results from the present study suggest that following surgically-induced knee derangement nociceptive neurons in the Aβ range may undergo important changes in physiology. Changes in other types of primary sensory neuron in this model are the subject of other studies.

## Methods

Experiments were done on female Sprague Dawley rats (180-225 g) obtained from Charles River Inc. (St. Constant, QC, Canada). All protocols were approved by the McMaster University Animal Review Ethics Board and all experimental procedures conformed to the Guide to the Care and Use of Laboratory Animals of the Canadian Council of Animal Care, Vols.1 and 2. Upon completion of the acute electrophysiological experiment each animal was euthanized by an overdose of anesthetic.

### Induction of the animal model of OA

The model of OA used was based on mechanical derangement of the knee [27]. For surgical induction of the model, animals were anesthetized with a mixture of ketamine (100 mg/ml), xylazine (20 mg/ml) and acepromazine (10 mg/ml) - ketamine from Bioniche (Belleville, ON, Canada), xylazine from Bayer (Toronto, ON, Canada), acepromazine from Wyeth-Ayerst (Guelph, ON, Canada). The joint capsule was exposed and the tibial and medial ligament attachments of the medial meniscus were severed to allow removal of the meniscus. The anterior cruciate ligament then became clearly visible and was cut. The incision was then sutured in two layers. Naive animals served as controls. Following the surgery, animals were sequentially given Trimel from Novopharm (Toronto, ON, Canada) 0.05 ml once per day for 3 consecutive days, and the analgesic, Temgesic from Schering-Plough (Kenilworth, NJ, USA), twice per day for 2 consecutive days.

We have previously found that in this model, mild cartilage degeneration, such as surface discontinuity, is observed 2-4 weeks after surgery [10], yet severe cartilage degeneration, such as vertical fissure formation and chondrocyte clusters, appears 8 weeks after surgery [10]. Therefore, 2-4 weeks after knee surgery in present OA model can be considered the initiation phase, whereas 8 weeks after surgery may represent a more advanced phase. To determine whether there would be temporal changes in electrophysiological properties of Aβ nociceptive primary sensory neurons that would correlate with these phases, acute electrophysiological recordings were carried out at two time points, early, at 4 weeks, and late, at 8 or more weeks following the surgery. Recordings were not made at 2 weeks after surgery in OA rats to avoid any acute surgical effect on neuronal properties.

### Animal preparation for acute electrophysiological recording

At one or two months after model induction each animal was initially anesthetized with the ketamine mixture described above. The right jugular vein was cannulated for i.v. infusion of drugs. An initial 1 mg/kg dose of pancuronium from Sandoz (Boucherville, QC, Canada) was given to eliminate muscle tone; the effect of pancuronium was allowed to wear off periodically (normally within one hour of pancuronium administration) in order to confirm a surgical level of anesthesia by observing the pupil for dilation and testing for reflex withdrawal from a pinch to a forelimb. Supplements of pentobarbital (CEVA SANTE ANIMALE, La Ballastière, Libourne, France; 20 mg/kg) and pancuronium (1/3 of the initial dose) were added every hour; this schedule of pentobarbital administration was confirmed to be effective in maintaining a surgical level of anesthesia in non-paralyzed control rats in our pilot study. An in-house servo-controlled infrared heating lamp maintained rectal temperature at approximately 37°C. The animals were mechanically ventilated (Model 683, Harvard Apparatus, QC, Canada); the ventilation parameters were adjusted to maintain the CO_2 _concentration at approximately 40 mmHg using end-tidal CO2 monitoring (CapStar-100 End-Tidal CO_2 _Analyzer, CWE, Ardmore, PA, USA).

The L4 DRG was selected for study. While L_3 _and L_4 _receive the most knee afferents [28] our pilot studies suggested that not only knee afferents were changed in this model, but changes were also in neurons innervating neighboring territories. In addition, in some cases it was important to stimulate the sciatic nerve and, as the majority of L_3 _afferents do not supply the sciatic nerve, the L4 DRG was selected for this study.

The rat was fixed in a stereotaxic frame and the vertebral column was rigidly clamped at the L_2 _and L_6 _vertebrae. The right femur was fixed by a customized clamp to avoid movements of the DRG during mechanical searching for peripheral receptive fields. Connective tissue over L_4 _DRG was removed with care. Exposed spinal cord and DRG were covered with warm paraffin oil to prevent drying. Direct heating of the DRG by the light source for the surgical microscope was carefully avoided. A pair of bipolar platinum stimulating electrodes (FHC, Bowdoinham, ME, USA) was placed beneath the L_4 _dorsal root that had been exposed and cut close to the spinal cord. The distance from the stimulation site (cathode) to the recording site (center of the DRG) was measured at the end of the experiment to determine the conductance distance and thereby calculate the conduction velocity of the fibers associated with each DRG neuron recorded. This conduction distance was normally between 12 and 16 mm.

### In vivo intracellular recording

The configuration of the AP is characteristic of each particular functional type of DRG neuron and thus can be used as one parameter for classification of each neuron recorded. For example, myelinated afferents that display a hump on the falling phase of the AP are considered to be nociceptors, while those that do not bear the hump innervate hairs, muscles, etc. and respond to innocuous stimulation [18,29]. Nociceptive neurons also exhibit other electrophysiological features, such as a broad AP duration, a relatively large AP amplitude and a long afterhyperpolarization duration [20]. Changes in the physiology of DRG neurons can be identified through changes in AP configuration. Thus, AP configuration was compared between control and model animals with the aim to identify changes in physiological properties of the neurons.

Thus, APs were obtained by intracellular recordings from somata in the DRG using micropipettes fabricated from filament-containing borosilicate glass tubing (1.2 mm outer diameter, 0.68 mm inner diameter; Harvard Apparatus, Holliston, MA, USA). The electrodes were pulled using a Brown-Flaming puller (Model P-87; Sutter Instrument Co., Novota, CA, USA) and filled with a 3 M KCl solution (DC resistance: about 40-70 M Ω).

During the acute electrophysiological experiment the microelectrode was advanced using an EXFO IW-800 micromanipulator (EXFO, Montreal, QC, Canada) until a hyperpolarization of at least -40 mV suddenly occurred and an AP could be evoked by stimulation of the dorsal root; APs were recorded with a Multiclamp 700B amplifier (Molecular Devices, Union City, CA, USA) and digitized on-line via a Digidata 1322A interface (Molecular Devices) with pClamp 9.2 software (Molecular Devices).

Measurement of electrophysiological parameters has been reported previously [19,30]. These include conduction velocity (CV), resting membrane potential (Vm), AP duration (APD), AP duration at half AP amplitude (AP half width), AP amplitude, AP rise time, AP fall time, maximum AP rising rate (MRR), maximum AP falling rate (MFR), afterhyperpolarization (AHP) amplitude, 50% AHP recovery time (AHP50) and 80% AHP recovery time (AHP80). Each of these parameters reflects a different mechanism contributing to the electrical properties of the neuron. Analysis was done offline using pClamp 9.2.

### Classification of dorsal root ganglion neurons

The criteria for neuron classification based on conduction velocity followed those reported in a previous *in vivo *study, in which Aβ-fiber conduction velocity was defined as greater than 6.5 m/s along the dorsal root in female Wistar rats [20]. We adopted this criterion because it most closely applied to the present studies compared with criteria from other labs [22,31,32], including the same gender (female), a comparable age at experiment (~160 g in Lawson's vs. ~250 g in ours), similar recording temperature due to similar surgical exposure, heating strategy and core temperature set-point.

The sensory receptive properties of DRG neurons were identified using specific mechanical stimuli, and classified as previously described [20] and as outlined below. High threshold mechanoreceptors (HTMs) were considered to be nociceptive neurons if they were activated by high intensity stimuli such as pinch or squeeze applied with a fine or coarse-toothed forceps, or a sharp object such as the tip of a syringe needle. Neurons included in this study did not show a response evoked by innocuous stimuli such as gentle pressure or brush with a camel hair brush.

Some neurons were the so-called "unresponsive neurons". These have been identified in earlier studies as those neurons that are not excited by any of the non-noxious or noxious mechanical stimuli listed above [33]. Among these, some might be nociceptive neurons based on the fact that they had a prominent inflection on the repolarization phase of the AP in differentiated recordings, which is considered to be a feature unique to nociceptive neurons [22,34] and which has been adopted as a criterion to differentiate nociceptive neurons from non-nociceptive neurons in *in vitro *electrophysiological studies where sensory property testing is not possible [18,22,35,36].

### Acceptance criteria

All neurons included in this study met the following criteria: they exhibited an evoked AP from dorsal root stimulation, had a Vm more negative than -40 mV and had an AP amplitude larger than 40 mV. In addition, for each neuron, before sensory testing was begun a continuous recording was obtained for ≥5 min after electrode penetration; only those neurons that maintained a stable Vm throughout recording and sensory testing are included in this report.

### Statistical analysis

The D'Agostino and Pearson omnibus test was carried out to determine normality of the electrophysiological data. In addition, wherever appropriate, one way analysis of variance (ANOVA) with Newman-Keuls post test or non-parametric Kruskal-Wallis test with Dunn's post test was used for comparison of parameters in control animals and at both stages of development of the OA model. *Fisher*'s exact test was used to analyze count data. Statistical tests and graphing were done using Prism 4 software (GraphPad, La Jolla, CA, USA), and *P *< 0.05 was considered to be significant.

## Results

All neurons included in this study were Aβ nociceptive neurons judged by sensory testing and by AP features. Electrophysiological properties of Aβ-fiber HTMs in control animals were comparable to those that have been reported from other research groups for this type of neuron [18-20,22]. Successful recordings that met the acceptance criteria were from a total of 23 neurons from 17 control animals and 47 neurons from 19 OA model animals. Following the criteria of Lawson et al. (1997) these were further differentiated into the following groups: Aβ-fiber nociceptor-like unresponsive neurons at one-month after model induction (*N *= 14), Aβ-fiber HTMs at one-month after model induction (*N *= 18) and Aβ-fiber HTMs at two months after model induction (*N *= 15). Very few Aβ-fiber nociceptor-like unresponsive neurons were observed in two-month OA animals. Therefore, no separate group was formed based on this type of neuron.

### Aβ-fiber nociceptor-like unresponsive neurons and Aβ-fiber HTMs

**Figure 1:**
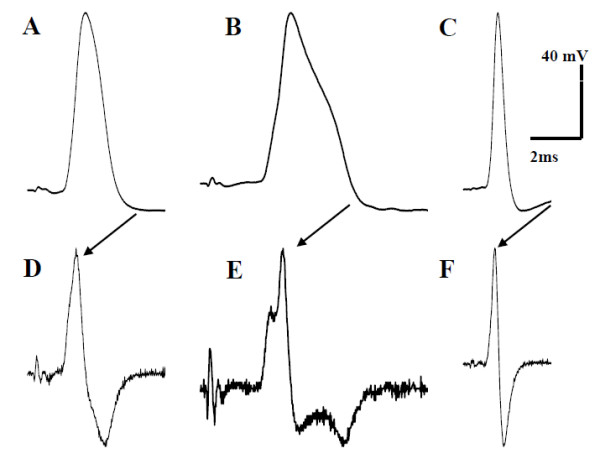
**Representative APs evoked in Aβ nociceptive neurons by dorsal root stimulation**. **A-C**, evoked APs; D-F, differentiated derivatives of these APs to determine rate of change. **A **and **D **are from a nociceptive neuron that could be activated only by high intensity stimuli, including firm pinch applied to the ankle joint; conduction velocity was 12.4 m/s. **B **and **E **are from an unresponsive neuron classified as an Aβ fiber on the basis of conduction velocity, which was 8 m/s, and a plateau was identified on the repolarisation branch of the AP in the differentiated recording, which is indicative of a nociceptive neuron. **C **and **F **are from another unresponsive neuron, with a conduction velocity of 18.8 m/s, but with no inflection on the falling phase in the differentiated recording, which is considered a non-nociceptive neuron.

Typical examples of an Aβ-fiber HTM and of an Aβ-fiber nociceptor-like unresponsive neuron are illustrated in Fig. 1. Note the prominent inflection observed in the representative Aβ-fiber nociceptor-like unresponsive neuron shown in Fig. 1B,E. This is consistent with earlier reports on this type of neuron from other laboratories and characterizes nociceptive neurons as described above [18,22,29,34].

The group of Aβ-fiber nociceptor-like unresponsive neurons might be heterogeneous having a mixture of nociceptive and non-nociceptive neurons. This is the only group that could have included hypothetical axotomized neurons. The proportion of nociceptor-like neurons in the total of unresponsive neurons was compared in the control vs. OA groups, and was 6 out of 21 (28.6%) in control and 14 out of 31 neurons (45.2%) in OA animals at one month; this seemly large difference in the proportion of Aβ-fiber nociceptor-like unresponsive neurons in OA was not significantly different (*P *= 0.26). We also calculated the proportion of "unresponsive nociceptors" in the nociceptor population between OA and control animals in case nociceptor-like unresponsive neurons are actual nociceptors. Again, there was no difference in this proportion: 6 out of 29, 20.7% in control vs. 14 out of 32, 43.8% in one month OA (P = 0.064). Thus, our observation was insufficient to substantiate a greater number of nociceptor-like unresponsive neurons in OA animals.

All Aβ-fiber HTMs in the present study were recorded from L_4 _DRG. Receptive fields of these neurons encompassed every major compartment of the ipsilateral lower limb: from knee joint (*N *= 3 each in control, OA at one month and OA at two months), from ankle joint (*N *= 3 in control, 2 in OA at one month, and 1 in OA at two months), from the leg (N = 5 in control, 1 in OA at one month, and 3 in OA at two months), from calf (*N *= 1 in control, none in OA at one month, and 1 in OA at two months), and from foot (*N *= 11 in control, 12 in OA at one month, and 4 in OA at two months). There were two general observations. First, most Aβ-fiber HTMs innervated deep tissues, such as joint, muscle and/or periosteum of the leg, and deep tissue of the foot. Second, the foot receives a rich innervation from Aβ-fiber HTMs with a comparable distribution in either hairy or glabrous skin.

### Changes in Aβ-fiber nociceptor-like unresponsive neurons and HTMs at one month

**Figure 2:**
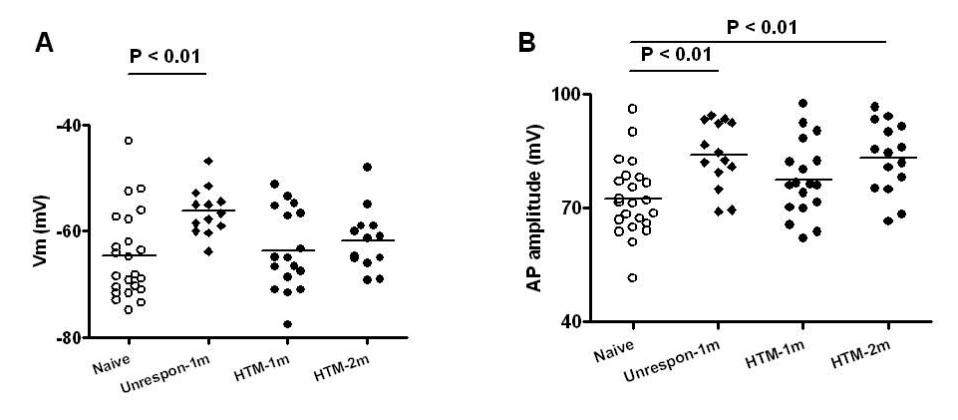
**Resting membrane potential (Vm) and action potential (AP) amplitude in Aβ nociceptive DRG neurons in osteoarthritis (OA) animals at one month and at two months, and in naïve control animals**. "Naive" represents Aβ-fiber HTMs in control animals. "Unrespon" represents nociceptor-like Aβ-fiber unresponsive neurons in one month OA animals; an inflection on the falling phase of the differentiated recording was used to classify these neurons [22,34]. "HTM-1m" and "HTM-2m" represent Aβ-fiber HTMs from the OA group tested one month or two months after model induction, respectively. One way ANOVA with Newman-Keuls post test was used for multiple comparisons among "Naive" (N = 23), "Unrespon" (N = 13 for Vm and N = 14 for AP amplitude), "HTM-1m" (N = 17 for Vm and N = 18 for AP amplitude), "HTM-2m" (N = 13 for Vm and N = 15 for AP amplitude). In each case the mean (horizontal line) is presented.

To examine the possible effects of direct nerve damage on AP configuration in the OA model, we compared Aβ-fiber nociceptor-like unresponsive neurons at one month OA with naïve control Aβ-fiber HTMs. Aβ-fiber nociceptor-like unresponsive neurons were the primary type significantly altered in AP configuration at one month. In these neurons, Vm was significantly depolarized compared with values from naïve controls (-56.3 ± 1.23 mV, *N *= 13 in OA and -64.7 ± 1.71 mV, *N *= 23 in control; *P *= 0.002; Fig. 2A). However, in this early phase of the model no change in Vm was observed in Aβ-fiber HTMs (Table 1).

**Table 1:** Properties of all the nociceptive DRG neurons recorded in control and osteoarthritis animals

**Parameter**	**Naïve**	**Unrespon-1m**	**HTM-1m**	**HTM-2m**
**Vm (mV)**	-64.66 ± 1.71, *N *= 23	-56.25 ± 1.23, *N *= 13	-63.65 ± 1.85, *N *= 17	-61.72 ± 1.62, *N *= 13

**Amplitude (mV)**	72.47 ± 2.04, *N *= 23	84.01 ± 2.33, *N *= 14	77.52 ± 2.35, *N *= 18	83.19 ± 2.4, *N *= 15

**APD (ms)**	1.56 ± 0.11, *N *= 23	1.79 ± 0.16, *N *= 14	1.68 ± 0.1, *N *= 18	1.13 ± 0.09, *N *= 15

**Half width (ms)**	0.78 ± 0.05, *N *= 23	1 ± 0.09, *N *= 14	0.86 ± 0.06, *N *= 18	0.56 ± 0.05, *N *= 15

**Rise time (ms)**	0.66 ± 0.06, *N *= 23	0.71 ± 0.06, *N *= 14	0.64 ± 0.04, *N *= 18	0.47 ± 0.04, *N *= 15

**MRR (mV/ms)**	239.1 ± 19.23, *N *= 23	228.8 ± 18.52, *N *= 14	239.1 ± 12.49, *N *= 18	350.4 ± 28.09, *N *= 15

**Fall time (ms)**	0.9 ± 0.05, *N *= 23	1.09 ± 0.1, *N *= 14	1.04 ± 0.06, *N *= 18	0.66 ± 0.05, *N *= 15

**MFR (mV/ms)**	135.7 ± 10.23, *N *= 23	121.1 ± 11.29, *N *= 14	124.3 ± 7.33, *N *= 18	197.5 ± 14.66, *N *= 15

**AHP (mV)**	10.7 ± 0.68, *N *= 23	11.78 ± 1.05, *N *= 13	12.19 ± 0.69, *N *= 15	10.11 ± 0.71, *N *= 14

**AHP80 (ms)**	30.95 ± 5, *N *= 22	35.9 ± 7.46, *N *= 13	34.71 ± 4.82, *N *= 15	21.01 ± 4.92, *N *= 14

**AHP50 (ms)**	8.99 ± 1.29, *N *= 22	9.6 ± 1.73, *N *= 13	8.67 ± 1.48, *N *= 15	5.71 ± 1.62, *N *= 14

**CV (mm/ms)**	14.1 ± 1, *N *= 23	11.69 ± 0.8, *N *= 14	13.24 ± 0.66, *N *= 18	16.38 ± 1.42, *N *= 15

"Naive" represents Aβ-fiber HTMs in control animals. "Unrespon-1m" represents nociceptor-like Aβ-fiber unresponsive neurons in one month OA animals. "HTM-1m" and "HTM-2m" represent Aβ-fiber HTMs from the OA group tested one month or two months after model induction, respectively. Values are presented as mean ± S.E.M. "*N*" represents the number of neurons.

AP amplitude is the net effect of depolarization and rectification forces, and was significantly less in nociceptive neurons of OA animals. At one month of the model no difference in AP amplitude was observed in Aβ-fiber HTMs. In Aβ-fiber nociceptor-like unresponsive neurons in OA animals, AP amplitude was 84.0 ± 2.33 mV (*N *= 14). This is 11.5 mV more hyperpolarized than that of the Aβ-fiber HTMs in naïve control animals, which was 72.5 ± 2.04 mV (*N *= 23; *P *= 0.001; Fig. 2B).

**Figure 3:**
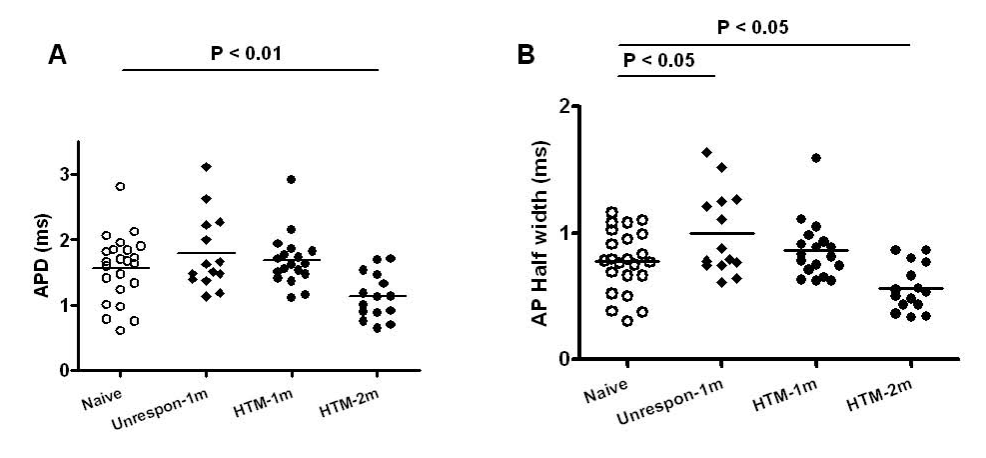
**Action potential duration at base (APD) and AP width at half amplitude (AP half width) in Aβ nociceptive DRG neurons in OA animals at one month and at two months, and in naïve control animals**. Labeling is otherwise the same as in Figure 2. One-way ANOVA with Newman-Keuls post test was used for multiple comparisons among groups as follows: "Naive" (N = 23), "Unrespon" (N = 14), "HTM-1m" (N = 18) and "HTM-2m" (N = 15).

APD reflects the net effect of overall ion flow. This did not differ in either type of nociceptive neuron between control and model animals at one month after model induction (Fig. 3A). Compared with the control animals, a significantly longer AP half width was found in Aβ-fiber nociceptor-like unresponsive neurons in OA model animals (1.0 ± 0.09 ms, *N *= 14 vs. 0.8 ± 0.05 ms, *N *= 23 in control; *P *= 0.027; Fig. 3B).

AP rise time is taken as a measure of the time for depolarization from baseline to peak and largely reflects Na^+ ^flux. No difference in AP rise time was found in Aβ-fiber HTMs or in Aβ-fiber nociceptor-like unresponsive neurons at one month after model induction (data not shown). MRR, used as an additional measure of the dynamics of depolarization, was derived by mathematical conversion of the AP waveform as the differentiated derivative of the AP. Thus, the curve represents the rate of voltage change over time. MRR reflects the maximum depolarization driving force, mostly generated by sodium influx current. MRR in Aβ-fiber HTMs in OA animals was not significantly different from control values (Table 1).

A similar rationale was adopted to determine the dynamics of repolarisation, where AP fall time and MFR were used as measures of the dynamics of the repolarisation phase of the AP. Significant differences in AP fall time and MFR were not seen between control and OA animals in Aβ-fiber HTMs or nociceptor-like unresponsive neurons (Table 1).

Basically, there are three types of AHP following the spike - a fast AHP (immediate activation during the spike having a duration of several tens of milliseconds), a medium AHP (immediate activation during the spike having a duration of several hundreds of milliseconds) and a slow AHP (slow activation over hundreds of milliseconds having a duration of several seconds) [37]. Different calcium-activated potassium channels underlie different AHPs, such as BK-type channels for fast AHP, SK-type channels for medium AHP [37]. The AHP80 in control Aβ-fiber HTMs was 30.9 ± 4.9 ms (*N *= 22), suggesting that the AHP in the present study was likely to be a fast AHP. However, none of the parameters of the AHP, including amplitude and duration, showed any difference between either OA Aβ-fiber HTMs or Aβ-fiber nociceptor-like unresponsive neurons and naive control Aβ-fiber HTMs, including duration or amplitude (Table 1).

Unlike the other parameters measured, which reflect properties of the soma, conduction velocity reflects properties of the axon. There were no statistically significant differences in conduction velocity between Aβ-fiber HTMs in controls vs. Aβ-fiber HTMs or Aβ-fiber nociceptor-like unresponsive neurons in OA animals (Table 1).

### Changes in Aβ-fiber HTMs at two months

At two months of model development, only a few Aβ-fiber nociceptor-like unresponsive neurons were recorded. Thus, the following data are from Aβ-fiber HTMs. Vm was similar in Aβ-fiber HTMs from control vs. model animals in this later phase of the model (Table 1). However, a significantly larger AP amplitude was seen in these neurons at this phase of model development (83.2 ± 2.40 mV, *N *= 15) compared to controls (72.5 ± 2.04 mV, *N *= 23; *P *= 0.002; Fig. 2B).

APD was significantly shorter in the OA model rats at two months compared with the control rats (1.1 ± 0.09 ms, *N *= 15 vs. 1.6 ± 0.11 ms, *N *= 23 in control; *P *= 0.007; Fig. 3A). AP half width was also significantly shorter in OA model rats in the late phase compared to control rats (0.6 ± 0.05 ms, *N *= 15 vs. 0.8 ± 0.05 ms, *N *= 23 in control; *P *= 0.007; Fig. 3B).

**Figure 4:**
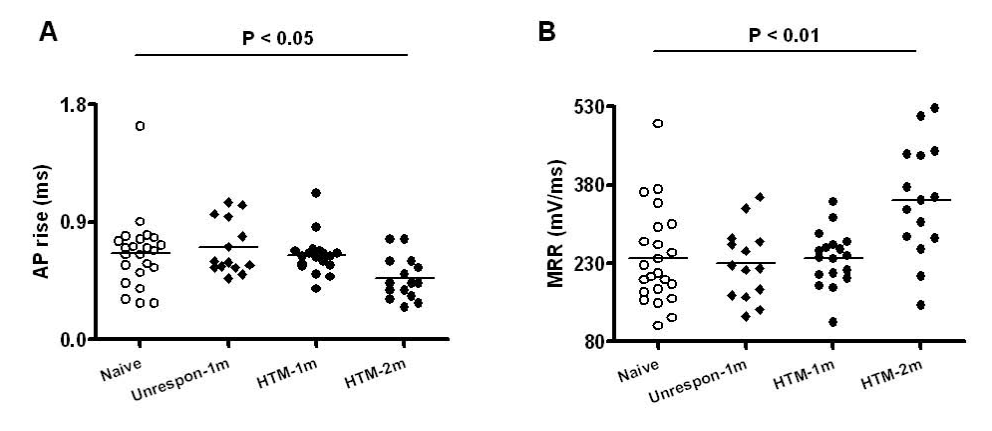
**Action potential rise time (AP rise) and maximum rising rate (MRR) in Aβ nociceptive DRG neurons in OA animals at one month and at two months, and in control animals**. Labeling is otherwise the same as in Figure 2. Kruskal-Wallis test with Dunn's post test was used for multiple comparisons among "Naive" (N = 23), "Unrespon" (N = 14), "HTM-1m" (N = 18) and "HTM-2m" (N = 15).

As shown in Fig. 4A, AP rise time was shorter in OA animals at two months of model development compared to control rats (0.5 ± 0.04 ms, *N *= 15 vs. 0.7 ± 0.06 ms, *N *= 23 in control; *P *= 0.021). MRR was 350.4 ± 28.09 mV/ms (*N *= 15) in Aβ-fiber HTMs in the late phase, which was significantly faster than the 339.1 ± 19.23 mV/ms (*N *= 23) in control rats (*P *= 0.002; Fig. 4B).

**Figure 5:**
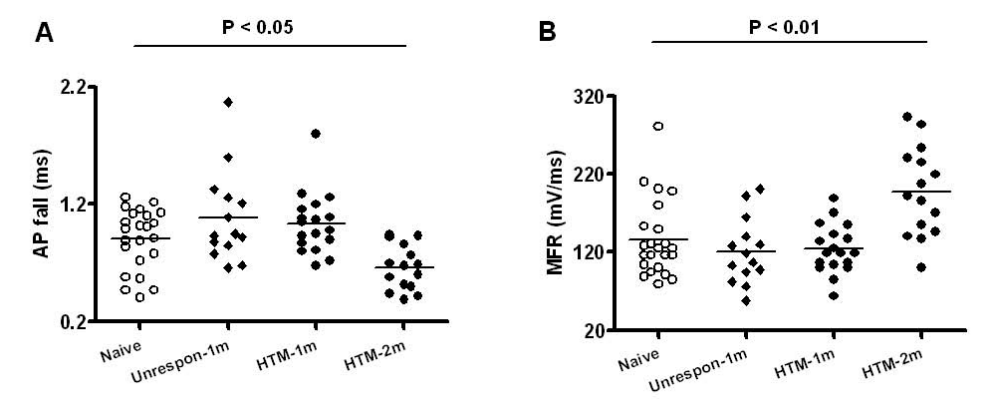
**Changes in action potential fall time (AP fall) and maximum falling rate (MFR) in Aβ nociceptive DRG neurons in OA animals at one month and two months, and in naïve control animals**. Labeling is otherwise the same as in Figure 2. Kruskal-Wallis test with Dunn's post test was used for multiple comparisons among the "Naive" (N = 23), "Unrespon" (N = 14), "HTM-1m" (N = 18) and "HTM-2m" (N = 15).

Similarly, as shown in Fig. 5A, a significantly shorter AP fall time was observed in Aβ-fiber HTMs at two months compared to control rats (0.7 ± 0.05 ms, *N *= 15 vs. 0.9 ± 0.05 ms, *N *= 23 in control; *P *= 0.004). Also, as shown in Fig. 5B, MFR was faster in the late phase OA animals compared to control rats (197.5 ± 14.66 mV/ms, *N *= 15 vs. 135.7 ± 10.23 mV/ms, *N *= 23 in control; P = 0.001).

In Aβ-fiber HTMs, there were no difference in conduction velocity or AHP associated parameters between control and OA animals at two months (Table 1).

## Discussion

The present study provides evidence that unilateral knee derangement induces changes in the AP recorded from Aβ-fiber nociceptive primary sensory neurons in the ipsilateral L4 dorsal root ganglion. Interestingly, the neuron types exhibiting changes differentiate into two groups, Aβ-fiber HTMs and Aβ-fiber nociceptor-like unresponsive neurons. These changes were also different in animals tested at two months after model induction vs. those tested at one month.

### Rationale for model selection and control

Several types of animal model of OA exist, including those induced by modulation of gene [38] or protein expression [39], injection of inflammatory cytokines [40] or injection of photolytic enzymes [41] as well as surgical induction [42] or excessive use of the joint [43]. The model selected for the present study is a surgically-induced derangement of the knee of one hind leg; while OA can originate from a number of causes, injury is the most common [26]. We have shown that our OA model successfully mimic changes in cartilage and bone closely matching the human condition, including cartilage edema and collagen turnover [7,8].

Controls for this study were naïve rats. Simple surgical exposure of the joint capsule, regardless of the size of the surgical exposure has been reported to cause joint instability [44] and articular cartilage degeneration [45], which would result in an unwanted comparison between severe and mild osteoarthritis. Even a surgical incision normally induces a brief inflammatory phase lasting only days [46,47], but is fully repaired with scar tissue devoid of inflammatory infiltration by the end of the first month [48].

### Changes in neuronal physiology in model animals at one month

The changes at one month were observed only in Aβ-fiber nociceptor-like unresponsive neurons bearing a hump on the repolarization branch. The changes observed reflect greater excitability, such as a relatively depolarized resting membrane potential and increased AP amplitude, as well as slowed dynamics of AP genesis, illustrated as longer AP half width. These changes might be able to be explained by nerve injury.

The in vivo intracellular recording technique is a sensitive means of identifying changes in intact neurons, but is less sensitive in differentiating between axotomised neurons from otherwise unresponsive neurons. If the receptive field of a neuron cannot be identified, there are two possibilities: either the neuron is axotomised or its receptive field is not sufficiently activated. The unresponsive neuron group might thus be heterogeneous. They could be neurons with a very high stimulation threshold, neurons with inaccessible receptive fields, neurons unexcitable from any receptive field, neurons that are only responsive to chemical stimuli [19], or neurons that have lost their receptive fields due to axotomy. Our OA model involves transection of the anterior cruciate ligament, which is innervated mostly by large size neurons from L7 (main sciatic nerve root) and L5-6 (main femoral nerve roots) in cats [49], as well as removal of the medial meniscus for which the anterior and posterior horns are highly innervated by several different mechanoreceptors [50,51]. As both structures are innervated, following surgery mechanical derangement of the joint may be accompanied by nerve trauma and may represent a source of pain [52].

Moreover, Aβ-fiber nociceptor-like unresponsive neurons were the only group that could include hypothetical axotomised nociceptors. The time course of the earliest irreversible stage of DRG neuron death following axotomy, peaks at two weeks, and continues to lead to the elimination of neurons, which peaks at one to two months [53]. The fact that this type of neuron was rarely encountered in model animals studied at two months after model induction in the present study (data not shown) might agree with the marked apoptosis of DRG neurons by two months [53].

### Changes in neuronal physiology in model animals at two months

The changes at two months after model induction were quite different from those at one month. The major changes reported here occur at two months or more after model induction; changes at one month are relatively minor compared to the later changes. Changes at this more advanced phase of OA reflected accelerated rather than slowed dynamics of AP genesis, including shorter APD and AP half width, shorter AP rise time and fall time, and faster MRR and MFR. The diverse changes in AP configuration in Aβ-fiber nociceptive neurons at different phases of OA might be attributed to transcriptional regulation of a variety of ion channels by neurotrophic factors [54] and/or other inflammatory mediators. Our previous microarray data indicate a dynamic change in gene expression during the progression of the model, which involves cytokine, chemokine, and growth factor signaling pathways [9]. However, detailed pathways leading to the specific changes are simply unknown at present. These changes in sensory neurons over such a prolonged period of time suggest that studies on sensory neuron changes in animal models should include later time points in model induction. Moreover, it is possible that these late-developing changes in sensory neurons may relate in some way to the types of pain associated with more advanced OA in humans [4].

## Conclusion

Results from the present study suggest that Aβ nociceptive neurons undergo changes in this surgically-induced model of OA. If these changes are representative of changes in injury-induced joint OA, these neurons may play an important role in OA pain. Importantly, there is a late onset of electrophysiological changes in these neurons, well beyond the time that changes in structure and in nociceptive scores appear, and these may relate to the episodes of intense pain that characterize advanced OA.

## Competing interests

The authors declare that they have no competing interests.

## Authors' contributions

JLH conceived of, designed, and coordinated the study. QW did the electrophysiological experiments, analyzed the data and performed statistical analyses. QW wrote the initial draft of the manuscript. Both authors worked on refining this draft and the revision based on editorial review. Both authors have read and approved the final manuscript.
